# Supplementary material for: Preoperative Factors Associated with Target Lesion Revascularization following Endovascular Therapy of the Superficial Femoral Artery
Source: J Clin Med. 2022 Aug 8;11(15):4606. doi: 10.3390/jcm11154606 (PMC9369774; doi:10.3390/jcm11154606)
Supplement: Supplementary file 1 [file jcm-11-04606-s001.zip › jcm-1779663-supplementary.pdf]

Table S1. Number and percentage of missing values in baseline characteristics, overall, and for both groups separately.

Variable	Total (n = 781)	No CD-TLR (n = 662)	CD-TLR (n = 119)
Gender	0 (0.0)	0 (0.0)	0 (0.0)
Date of intervention	0 (0.0)	0 (0.0)	0 (0.0)
Age at first intervention	0 (0.0)	0 (0.0)	0 (0.0)
BMI	25 (3.2)	25 (3.8)	0 (0.0)
Diabetes	7 (0.9)	7 (1.1)	0 (0.0)
Hypertension	4 (0.5)	4 (0.6)	0 (0.0)
CAD	7 (0.9)	7 (1.1)	0 (0.0)
CVD	5 (0.6)	5 (0.8)	0 (0.0)
Dyslipidemia/statin/ezetimib	10 (1.3)	10 (1.5)	0 (0.0)
Number of interventions (within one year)	0 (0.0)	0 (0.0)	0 (0.0)
Smoking status	21 (2.7)	21 (3.2)	0 (0.0)

Table S2. Number and percentage of missing values in laboratory parameters for both groups within a time window of 30, 90, or 180 days after the intervention.

Variable	Single Intervention			Several Interventions		
	30 Days	90 Days	180 Days	30 Days	90 Days	180 Days
Cholesterol	121 (18.3)	111 (16.8)	103 (15.6)	18 (15.1)	12 (10.1)	9 (7.6)
Creatinine P	4 (0.6)	4 (0.6)	5 (0.8)	1 (0.8)	1 (0.8)	0 (0.0)
CRP	405 (61.2)	369 (55.7)	340 (51.4)	75 (63.0)	62 (52.1)	51 (42.9)
eGFR	4 (0.6)	4 (0.6)	5 (0.8)	1 (0.8)	1 (0.8)	0 (0.0)
HbA1c	137 (20.7)	134 (20.2)	117 (17.7)	19 (16.0)	17 (14.3)	13 (10.9)
HDL-C	120 (18.1)	111 (16.8)	104 (15.7)	19 (16.0)	13 (10.9)	9 (7.6)
LDL-C	121 (18.3)	111 (16.8)	104 (15.7)	19 (16.0)	13 (10.9)	9 (7.6)
Triglyceride	122 (18.4)	112 (16.9)	105 (15.9)	18 (15.1)	12 (10.1)	9 (7.6)

eGRF = estimated glomerular filtration rate (calculated); CRP = C-reactive protein; LDL = low-density lipoprotein cholesterol; HDL = high-density lipoprotein cholesterol; BMI = body mass index.
